# Efficacy of airway stenting and nasogastric tube insertion in airway–esophageal fistula patients with airways compromised by advanced malignancy

**DOI:** 10.1111/crj.13737

**Published:** 2024-02-13

**Authors:** Xue Wu, Guangbing Lu, Lin cheng Luo, Hailong Wei, Qun Yi, Wei Luo

**Affiliations:** ^1^ Department of Respiratory and Critical Care Medicine The Affiliated Hospital of Southwest Medical University Luzhou China; ^2^ Department of Respiratory and Critical Care Medicine The People's Hospital of Leshan Leshan China; ^3^ Department of Critical Care Medicine Meishan Traditional Chinese Medical Hospital Meishan China; ^4^ Department of Respiratory and Critical Care Medicine West China Hospital of Sichuan University Chengdu China; ^5^ Department of Critical Care Medicine Sichuan Cancer Hospital Chengdu China

**Keywords:** airway stent, airway–esophageal fistulas, malignancy, survival analysis

## Abstract

**Introduction:**

Whether airway‐compromised airway–esophageal fistula (AEF) patients should undergo combined airway and esophageal stenting is controversial. This study was designed to evaluate the survival prognosis and poststent interventions in AEF patients with airways compromised by advanced malignancy with or without airway stents.

**Methods:**

A retrospective analysis of the medical records, survival times, and poststent interventions of 17 patients with or without airway stents was performed.

**Results:**

The causes of AEF were esophageal cancer (11/17, 64.7%), lung cancer (6/17, 29.4%), and thyroid cancer (1/17, 5.9%). All patients received a nasogastric tube (*n* = 12) or underwent gastrostomy (*n* = 5) to resume enteral nutrition. Thirteen patients underwent airway stent insertion (13/17, 76.5%), whereas four patients did not. Four patients with a high risk of stent migration received external stent fixation to the trachea. Three of the patients with stents suffered severe granulation tissue formation and needed repeated bronchoscopy interventions. In the stented group, none of the patients developed stent migration, and the overall median survival time was 9 months, compared with 1.25 months in the nonstented group (*P* = 0.04). Cox proportional hazards regression revealed that stent insertion, nasogastric tube insertion, and transcatheter bronchial artery chemoembolization were protective factors against death, whereas surgery‐related fistula, fistula larger than 2 cm, continued chemotherapy, and age were risk factors for poor survival (*P* < 0.05).

**Conclusion:**

In airway‐compromised AEF patients, airway stents and nasogastric tubes are probably the preferred treatments. Airway stenting is tolerable, and routine weekly poststent bronchoscopy is needed in the first month and depending on respiratory symptoms thereafter.

## Graphical abstract

In airway‐comprised AEF patients with stent insertion, the median overall survival time was 9 months, which was 2 months inpatient without stent. In airway‐comprised AEF patients, airway stents and nasogastric tubes are the preferred treatments and the role of esophageal stents is not as important as previously stated.

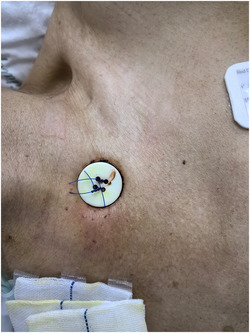

## 1 INTRODUCTION

Airway–esophageal fistula (AEF) can occur secondary to invasion by locally advanced esophageal, thyroid, or lung cancer; tumor necrosis due to radiation therapy (most common); chemotherapy; or complications from esophageal stenting. The prognosis of AEF is very poor, and without any intervention, the natural medical course is only a few weeks.^1^ Generally, the purpose of treatment in patients with AEF is to seal the defect to prevent aspiration and consequent pneumonia and improve nutritional status. Some expert consensuses have proposed that esophageal stenting alone should be initially pursued, with airway stenting considered under certain circumstances, such as when there is preexisting airway compromise, when the results of esophageal stenting are not satisfactory, or when the esophageal stent causes airway compression.^2^, ^3^, ^4^, ^5^ Airway compromise was defined as anything that prevented the movement of oxygenated air into and out of the lungs, including airway stenosis, compression, secretions, swelling, or other reasons for impaired ventilation. Airway‐compromised AEF patients should undergo combined airway and esophageal stenting. Herth's study suggested that combined stenting led to a longer survival time than airway stenting alone.^6^ Roseira et al. reached a similar conclusion.^7^ However, Pei‐Ming Huang et al. found no significant difference in survival time (*P* = 0.222) between the single‐stenting group and dual‐stenting group.^8^ Combined stenting leads to a high risk of AEF enlargement, and esophageal stents have a high risk of migration 4 weeks after placement, which can cause ineffective closure of the AEF.^9^ Moreover, the use of one additional stent means that the cost is greater and that there is more discomfort during poststent intervention. Here, we propose a substitutional treatment mode for combined stenting by using a nasogastric tube instead of an esophageal stent.

We focused on the efficacy and safety of the airway stent plus nasogastric tube protocol in airway‐compromised AEF patients, which meant that not only survival outcomes but also poststent complications and interventions were closely monitored in this group of patients.

## 2 MATERIALS AND METHODS

This retrospective study was approved by the Institutional Review Board of the People's Hospital of Leshan, and the requirement for informed consent was waived.

### 2.1 Study population

Seventeen patients with AEF were enrolled from September 2019 to December 2022. AEF was diagnosed by bronchoscopy, esophagoscopy, or barium/gastrografin esophagography. Moderate to severe airway stenosis or airway compromise was present in all patients.

### 2.2 Airway stenting procedure

**FIGURE 1:**
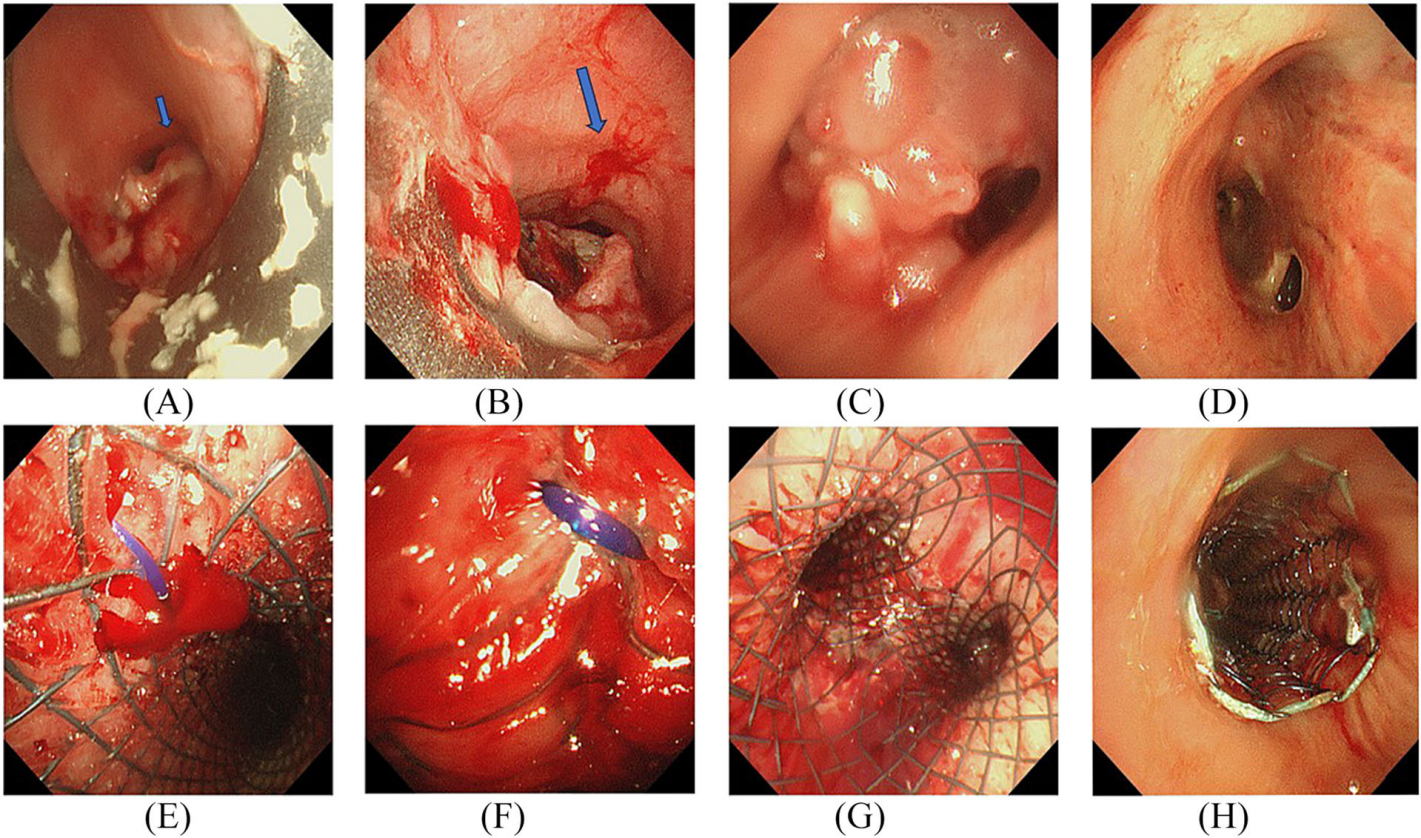
Different fistula locations and stent types. (A) Fistula located in the upper part of the trachea and beyond the cricoid (blue arrow). (B) Fistula located in the upper part of the trachea and beyond the cricoid (blue arrow). (C) Fistula located in the left bronchus and neoplasm near the carina. (D) Fistula related to surgery located in the right bronchus. (E) Straight stent fixed to the trachea (responses to A). (F) Straight stent fixed to the trachea (responses to B). (G) A Y‐shaped stent was inserted (response to C). (H) Straight stent inserted in the right bronchus (responses to D).

All procedures were performed under general anesthesia and with rigid bronchoscopy (Karl Storz, Tuttlingen, Germany). Airway stents (Boston Scientific Corporation, Boston, MA, USA) and Nanjing Microtech (Nanjing, China) were used for all patients. The decision regarding stent type was based on the location of the TEF; straight stents were inserted into the trachea and main bronchus, and the Y type was chosen for insertion near the carina. The stent was 10%–20% larger in diameter than the internal airway adjacent to the fistula orifice. To avoid stent displacement, approximately 5 mm of both the upper and lower margins of the stents usually remained uncovered. When straight stents had a high risk of migration, external fixation to the trachea was applied.^10^ We defined high risk of migration as the position of the upper margin of the stent being beyond the cricoid or even under the subglottic after deployment (Figure 1A,B). Complications, including timely elimination of secretions, repositioning of the stent after migration, and treatment of granulation tissue in the first month, were closely monitored. All patients were nebulized with 20% N‐acetylcysteine and albuterol twice daily to prevent mucus plugging in the first week.

### 2.3 Outcome measures

The primary outcome was overall survival. The secondary outcomes were quality of life (QoL) improvement and complications. The European Organization for Research and Treatment of Cancer QoL questionnaire (EORTC QLQ‐C30, version 3) was developed to assess the QoL of cancer patients.^11^ The QoL questionnaire was administered to all patients prior to and 6 weeks after stenting. The follow‐up duration was defined as the period from the date of stent implantation until the date of death, the last follow‐up date (30 December 2022), or the last day available for follow‐up.

### 2.4 Covariates

A total of 24 variables were extracted, including patient‐related variables, treatment‐related variables, and lesion‐related variables. The patient‐related variables included patient demographics, dyspnea index, survival time, parenteral nutrition, and diagnosis. The lesion‐related variables were as follows: location (divided into upper/lower trachea and right/left bronchus); size (fistula larger than 2 cm); presence of stent‐related mucus plugging; and presence of severe granulation tissue (defined as granulation tissue obstructing half of the lumen or more). Treatment‐related variables included treatment mode before the fistula, surgery, chemotherapy, radiotherapy, nasogastric tube or gastrostomy, stent fixation, stent dislocation, and continued chemotherapy after stenting.

### 2.5 Statistical analysis

Student's *t*‐test and *χ* ^2^ analysis were performed to detect differences in continuous variables and categorical variables between the two groups, respectively. Survival outcomes were estimated by the Kaplan–Meier method. Univariate and multivariate analyses by Cox proportional hazards regression were carried out to identify possible predictors of survival events (variables with a *P‐*value of <0.2 in the first step were included in the next step). A *P*‐value of <0.05 was defined as statistically significant. Statistical software (R 4.2; IBM Corp., Armonk, NY) was used for the data analysis.^12^

## 3 RESULTS

### 3.1 Patient characteristics

Our study cohort consisted of 14 men and three women aged between 49 and 77 years, with a mean age of 62.82 ± 7.55 (M ± SD) years. Thirteen patients received stent insertion, whereas four did not. The underlying malignant diseases were esophageal cancer (11/17, 64.7%), lung cancer (5/17, 29.4%), and thyroid cancer (1/17, 5.9%). The follow‐up period ranged from 0.3 to 15 months, with a mean of 6.06 ± 5.48 months and a median of 3 months. At the end of the follow‐up, two patients were still alive. In the stented group, 12 patients were men (12/13, 92.3%), and the mean age of all patients in the group was 62.3 ± 6.75 years, ranging from 49 to 77 years. All patients were asked to fast until nasogastric tube placement (*n* = 12) or gastrostomy (*n* = 5), after which they resumed enteral nutrition. In the no‐stent group, two patients were men (2/4, 50%), and the mean age was 64.5 ± 7.55 years, ranging from 58 to 73 years. Among the 13 stents, six were Y‐shaped and the rest were straight (Figure 1A,B,C,E,F,G).

Of the seven straight stents, four were fixed to the trachea, and no straight stents became dislocated or needed their location adjusted. All stented patients suffered from mucus plugging to different extents. Two patients underwent transcatheter bronchial artery chemoembolization (TACE) to avoid bleeding before the stent insertion procedure. Eight patients underwent surgery, 10 patients received chemotherapy, and six patients received radiotherapy before the fistula emerged. All patients suffered from dyspnea, and the mean dyspnea index^13^ was 3.88 ± 1.41, which decreased to 2 ± 1.13 after stent implantation (*P* < 0.05). Four patients continued to receive chemotherapy after stent implantation.

### 3.2 QoL score

**FIGURE 2:**
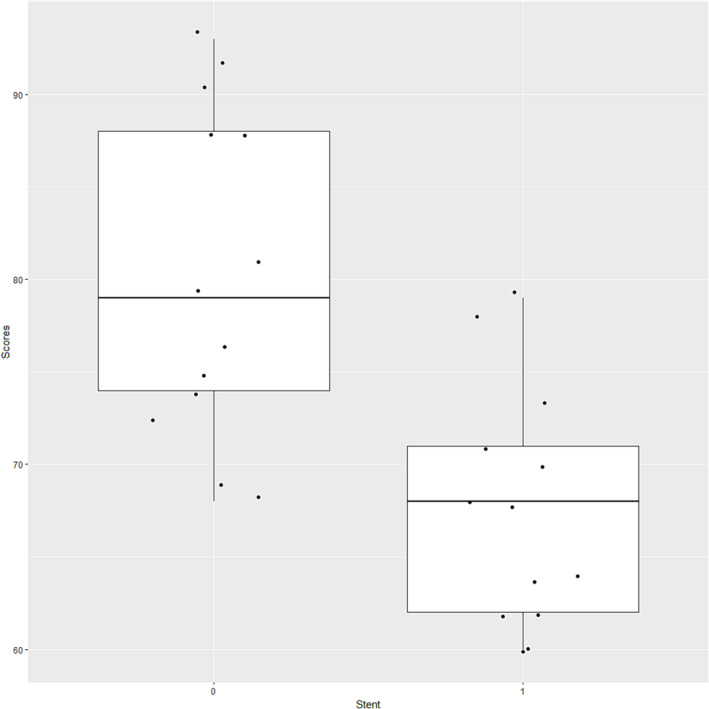
Analysis of quality of life (QoL) scores. The box plot shows the mean QoL scores prestent and poststent insertion.

The QoL scores before and after stent insertion are shown in Figure 2 (11 patients excluding two surgery‐related fistula patients). QoL score improved at 6 weeks postinsertion. There was also marked improvement in overall health and QoL (paired *t*‐test *P* < 0.05). A lower score meant a better QoL (Figure 2).

### 3.3 Poststent interventions and complications

None of the patients died because of the stent insertion procedure, and none of the stents were removed because of complications. There were no complications of perforation or mediastinitis, and none of the patients developed stent migration or needed stent repositioning. All patients exhibited different extents of poststent granulation tissue formation, among whom three patients suffered from severe poststent granulation tissue formation, which resulted in severe obstruction of the airway and necessitated repeated ablation and cryotherapy. The stent was removed within 4–6 weeks. All stented patients needed routine bronchoscopy weekly to clear mucus plugs in the first month. The need to continue bronchoscopy depended on the patients' clinical symptoms. The nasogastric tube was changed monthly.

### 3.4 Survival analysis (predictors of postintervention prognosis)

**FIGURE 3:**
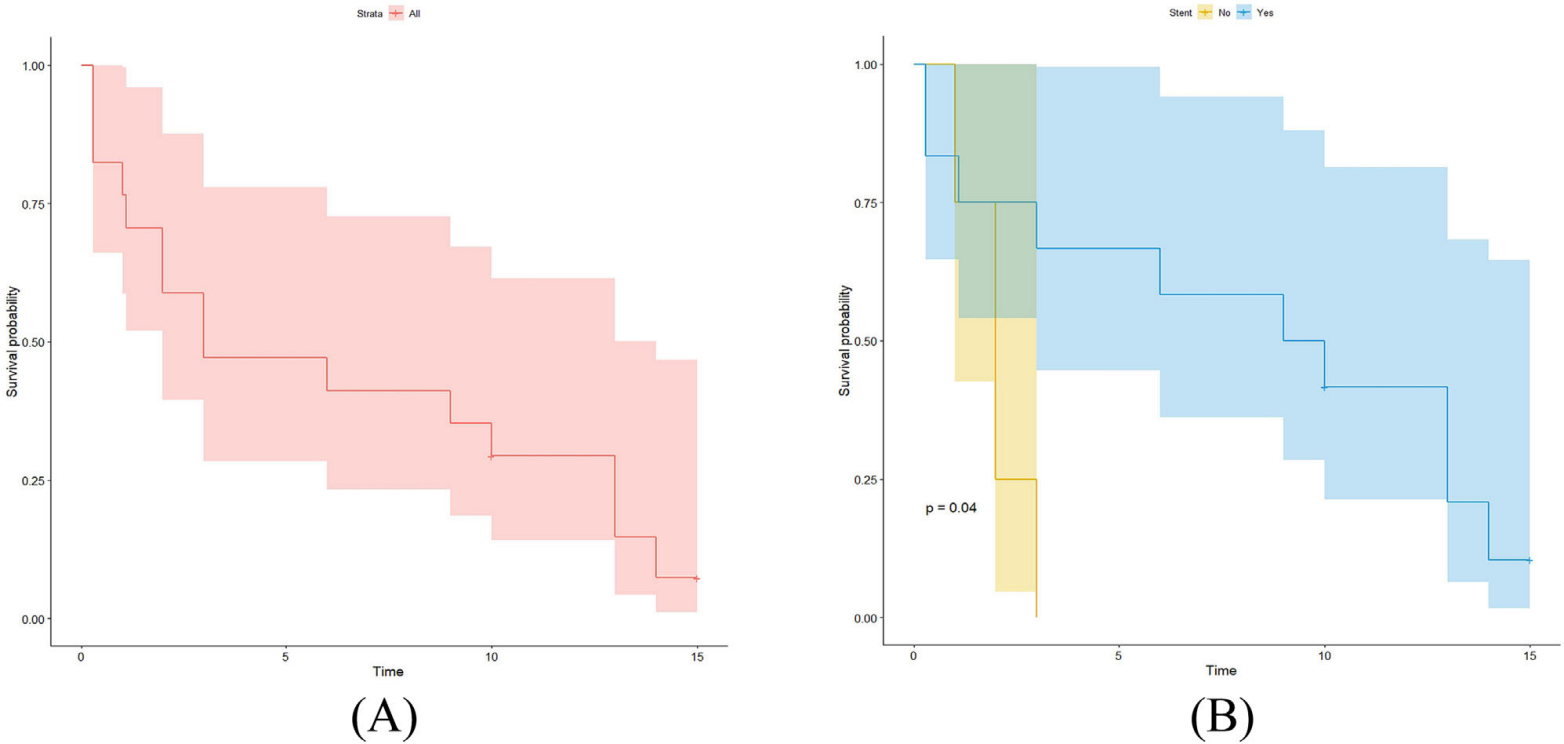
Analysis of survival prognosis. (A) Cumulative survival rates in all patients, calculated by the Kaplan–Meier method (colored area represents 95% confidence interval). (B) Kaplan–Meier estimated curves of survival rate in the stent and nonstent groups separately (the colored area represents 95% confidence interval).

Among all the patients, the overall median survival time was 3 months, the shortest survival time was 10 days, and the longest survival time was 15 months after the fistula emerged. In the stented group, the median survival time was 9 months, whereas in the nonstented group, the median survival time was 1.25 months (*P* = 0.04) (Figure 3A,B).

Possible predictors of prognosis in the 17 patients are shown in Table 1. Patients with surgery‐related fistula had the worst prognosis (Figure 1D,H); two patients died within 2 weeks after stent implantation (hazard ratio 25, 95% confidence interval [2.1 to 290], *P* = 0.01); and other significant correlations were found, including a fistula larger than 2 cm and severe granulation tissue formation according to the univariate analysis (Table 2).

**TABLE 1:** Baseline characteristics of all patients and between the stent and the nonstent group.

Patients characteristics	All patients*N* = 17	Stent*N* = 13	Nonstent*N* = 4	*P*‐value
Male/Female ratio	14/3	12/1	2/2	0.23
Age (Years)	62.82 ± 7.55	62.3 ± 6.75	64.5 ± 7.55	0.62
Stent type (Model Y/Straight)	6/7	6/7	0	NA
Diagnose (lung cancer/esophageal cancer)	6/11	5/8	1/3	0.81
Radiotherapy	6	4	2	0.63
Surgery before fistula	8	5	3	0.48
Chemotherapy before fistula	10	6	4	0.56
Dyspnea index before stent	3.88 ± 1.41	3.54 ± 1.75	5 ± 2.15	0.37
Fistula larger than 2 cm	7	6	1	0.68
Surgery‐related fistula	2	2	0	1
TACE	2	2	0	1
Death	15	11	4	1
Living time	6.06 ± 5.48	7.31 ± 6.15	1.25 ± 1.03	0.05
Stent fixation	4	4	NA	NA
Dyspnea index after stent	2.54 ± 1.15	1.54 ± 1.15	5 ± 2.15	0.05
Poststent moderate to severe granulation formation	3	3	NA	NA
Stent sputum stasis	2.59 ± 1.33	2.59 ± 1.33	NA	NA
Stent dislocation	0	0	NA	NA
Nasogastric tube	12	9	3	0.89
Gastrostomy	5	4	1	1
Continue chemotherapy	4	4	0	1
Performance status	3.25 ± 1.81	2.75 ± 1.31	3.45 ± 1.61	0.28
Lesion location				
Upper trachea	9	7	2	0.68
Lower trachea	2	2	0	0.54
Left bronchus	4	3	1	0.9
Right bronchus	2	1	1	1

*Note*: Data are mean ± standard deviation.

**TABLE 2:** Univariate and multivariate Cox regression analyses of predictive factors for survival outcome.

Variables	Univariate analysisHR (95% CI) *P*‐value	Multivariate analysisHR (95% CI) *P*‐value
Male/Female ratio	0.56 (0.15–2.1) 0.39	
Age (Years)	1.1 (0.99–1.2) 0.07	1.3e+00 (1.0e+00 1.7e+00) 0.029
Stent type (Model Y/Straight)	0.89 (0.3–2.6) 0.84	
Diagnose (lung cancer/esophageal cancer)	0.38 (0.45–4.7) 0.81	
Radiotherapy	0.58 (0.25–1.7) 0.39	
Surgery before fistula	2.6 (0.87–7.6) 0.08	
Chemotherapy before fistula	1.8 (0.61–5.3) 0.29	
Dyspnea index before stent	0.83 (0.53–1.3) 0.4	
Fistula larger than 2 cm	0.28 (0.08–0.98) 0.05	3.0e+03 (1.2e+01 7.6e+05) 0.005
Surgery related fistula	25 (2.1–290) 0.01	2.4e+08 (1.6e+03 3.6e+13) 0.001
TACE	0.19 (0.024–1.5) 0.11	7.0e‐04 (6.5e‐07 7.6e‐01) 0.042
Survival time	1.8e‐40 (0‐Inf) 0.92	
Stent fixation	0.59 (0.19–1.9) 0.38	
Dyspnea index after stent	1.2 (0.62–2.2) 0.64	
Poststent moderate to severe granulation formation	0.31 (0.068–1.4) 0.03	1.6e+02 (2.1e+00 1.1e+04) 0.021
Stent sputum stasis	0.59 (0.36–0.94) 0.12	
Stent dislocation	1 (0.13–8) 0.99	
Nasogastric tube	0.31 (0.089–1.1) 0.07	8.2e‐03 (2.5e‐04 2.7e‐01)0.007
Gastrostomy	1.5 (0.49–4.5) 0.48	
Stent	0.31 (0.078–1.2) 0.08	2.5e‐04 (2.9e‐07 2.1e‐01) 0.016
Continue chemotherapy	0.38 (0.11–1.4) 0.14	3.2e+01 (1.0e+00 1.0e+03) 0.049
Performance status	1.6 (0.61–2.3) 0.29	
Lesion location		
Upper trachea	1.3 (0.61–1.3) 0.69	
Lower trachea	0.8 (0.41–3.3) 2.34	
Left bronchus	1.2 (0.78–4.3) 0.32	
Right bronchus	2.2 (0.38–6.3) 0.75	

Abbreviations: CI, confidence interval; HR, hazard ratio; Inf, infinitely.

**FIGURE 4:**
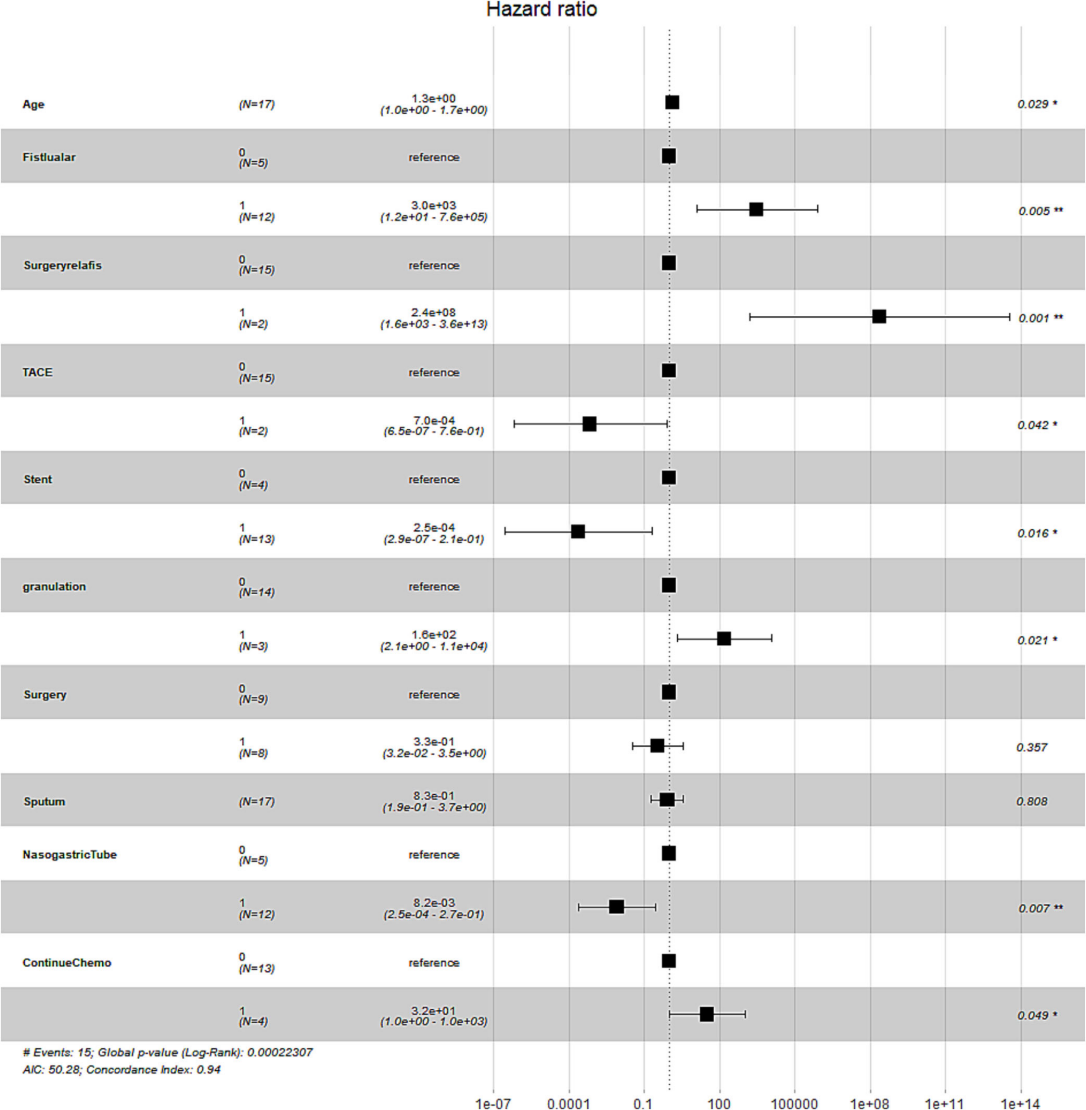
Multivariate Cox regression plot of hazard ratios and 95% confidence intervals. fistular, fistula larger than 2 cm; surgeryrelafis, surgery‐related fistula; continuechemo, continue chemotherapy; sputum, sputum plugging; TACE, transcatheter bronchial artery chemoembolization.

The multivariate Cox regression analysis included 10 variables that had a *P*‐value of <0.2 in the univariate analysis. Eight significant correlations were found among these factors (Table 2): Stenting, nasogastric tube insertion, and TACE were protective factors, whereas surgery‐related fistula, fistula larger than 2 cm, severe granulation tissue formation, continued chemotherapy, and age were risk factors (Figure 4).

## 4 DISCUSSION

Regarding the treatment of AEF patients, all of the recommendations^3^, ^4^, ^5^ were based mainly on the study by Herth,^6^ which indicated that the survival prognosis of patients receiving esophageal stents or combined esophageal and airway stents was better than that of patients who only received airway stents. In Herth's research, esophageal stents led to longer overall survival (OS) than combined esophageal and airway stents or airway stents only (mOS 262.8 vs. 252.9 vs. 219.1 days) in AEF patients. However, the reason why airway stents are associated with a worse survival prognosis is not clear, and only 10 AEF patients were included in this subgroup. The author proposed that the airway‐stented group likely had more advanced disease. Freitag et al. studied 18 AEF patients who underwent combined stenting and achieved an mOS of 110 days.^14^ Khan et al. described 29 AEF patients with an mOS of 97 days (range, 17 to 297 days) after dual stenting.^15^ Some other studies have suggested that combined stenting could improve survival time compared with airway stenting alone.^7^, ^8^, ^16^ Yonghua Bi et al. reported that 35 consecutive patients underwent combined stenting with a relatively long OS and that the 1‐, 3‐, and 5‐year survival rates were 82.4%, 78.8%, and 78.8%, respectively, in a retrospective analysis.^17^ However, there were two limitations to their study. First, they claimed 35 patients were enrolled, but 54 patients were analyzed and the summed ratio was 100.3% in their table. Second, there was no subgroup analysis of survival prognosis in the AEF patients. In summary, most of those studies did not demonstrate marked improvements in mOS (mOS only around 100 days) in the combined stenting group (Table 3).

**TABLE 3:** Summary of AEF patients with double stents studies.

Year	Author	Study type	Cohort study	Number of AEF patients	Combined stenting	mOS (days)	Reference
1992	Henri G. Colt et al.	Not mentioned	Yes	5	Yes	Not mentioned	^16^
1996	Lutz Freitag et al.	Retrospective	Yes	18	Yes	110	^14^
2010	Herth et al.	Prospective	Yes	10	Yes	252.9	^6^
2016	Pei‐Ming Huang et al.	Retrospective	Yes	16	Yes	Not mentioned	^8^
2019	J. Roseira et al.	Prospective	No	36	Yes	Not mentioned	^7^
2020	Ajmal Khan et al.	Prospective	No	29	Yes	97	^15^

In airway‐compromised AEF patients, airway obstruction, pulmonary infection, and poor nutritional status are significant life‐threatening complications, among which airway obstruction requires urgent correction. Under these conditions, reestablishing airway patency was more important than restoring the seal of the fistula. Nasogastric tubes were better than gastrostomy/jejunostomy for replacing esophageal stents for the purpose of resuming enteral feeding and supplying enough nutrition. All of the recruited patients had advanced‐stage disease, and most patients received chemotherapy and surgery before AEF emerged. In the airway‐stented group, the mOS was 9 months, which was much better than the 219.1 days in Herth's trial. Multivariate analysis demonstrated that patients could benefit from airway stents, nasogastric tubes, and TACE. Continued chemotherapy after stenting, severe granulation tissue formation, a fistula larger than 2 cm, and surgery‐related fistula were risk factors for death. Among these risk factors, surgery‐related fistulas had the worst prognosis, and two patients died 2 weeks after surgery because of severe secondary lung infection and subsequent complications. Severe granulation tissue formation is a severe complication after stenting that can lead to less relief of dyspnea and more bronchoscopic interventions. A fistula larger than 2 cm usually indicates a long course of disease, and these patients have a serious condition. In the AEF stage, chemotherapy is no longer recommended. In the nonstented group, even with best supportive care, the mOS was 1.25 months, which was slightly better than the natural course of disease.^1^ The most common complications of straight stents were mucus plugging and granulation tissue formation, which made bronchoscopic intervention necessary in the first month. We did not observe stent migration in the stented group, which was reported to be the major complication.^9^ Among the seven straight‐stent patients, only four underwent stent fixation, which means that not all straight‐stent patients need this invasive procedure. In addition to strictly following the rule of choosing the stent mentioned before, stent fixation should be carried out when the stent tends to migrate.

In addition to the survival benefit, the overall QoL score improved significantly after stent insertion. This means that even when encountering poststenting problems, including painful bronchoscopic experiences, uncomfortable nasogastric tube fixation on the face, and the inability to taste food, patients still benefit greatly from this treatment mode.

Our study demonstrated that the protocol of airway recanalization and resumption of enteral feeding in airway‐compromised AEF patients is a new treatment option. Its benefits in survival time, QoL, trauma, and costs make the role of esophageal stents in AEF patients probably not as important as previously stated.

In conclusion, for airway‐compromised AEF patients, airway stents and nasogastric tubes are the preferred treatment choices. The complications of airway stents are acceptable, and routine weekly poststent bronchoscopy is needed in the first month, with the requirements depending on the respiratory symptoms thereafter.

There were some limitations to our study. First, this was a retrospective study with inherent limitations. Second, a relatively small number of patients were enrolled.

## AUTHOR CONTRIBUTIONS

**Wei Luo:** Conceptualization; investigation; methodology; project administration; supervision; carrying out the stent‐insertion; review and editing manuscript. **Qun Yi:** Conceptualization; methodology; supervision; review and editing manuscript. **Xue Wu:** Data collection; formal analysis; funding acquisition; writing original draft. **Guangbing Lu:** Data collection; formal analysis; funding acquisition; project administration. **Lin cheng Luo:** Data curation; investigation; project administration; carrying out the stent‐insertion. **Hailong Wei:** Data curation; investigation; project administration.

## CONFLICT OF INTEREST STATEMENT

None.

## ETHICS STATEMENT

The retrospective study was approved by the Institutional Review Board of the People's Hospital of Leshan.

## PATIENT CONSENT STATEMENT

The retrospective study was waived for informed consent.

## PERMISSION TO REPRODUCE MATERIAL FROM OTHER SOURCES

None.

## CLINICAL TRIAL REGISTRATION

Not applicable.

## Data Availability

The data that support the findings of this study are available from the corresponding author upon reasonable request.
